# Revision of the Li_13_Si_4_ structure

**DOI:** 10.1107/S1600536813029759

**Published:** 2013-11-06

**Authors:** Michael Zeilinger, Thomas F. Fässler

**Affiliations:** a Department of Chemistry, Technische Universität München, Lichtenbergstrasse 4, 85747 Garching, Germany

## Abstract

Besides Li_17_Si_4_, Li_16.42_Si_4_, and Li_15_Si_4_, another lithium-rich representative in the Li–Si system is the phase Li_13_Si_4_ (trideca­lithium tetra­silicide), the structure of which has been determined previously [Frank *et al.* (1975 ▶). *Z. Naturforsch. Teil B*, **30**, 10–13]. A careful analysis of X-ray diffraction patterns of Li_13_Si_4_ revealed discrepancies between experimentally observed and calculated Bragg positions. Therefore, we redetermined the structure of Li_13_Si_4_ on the basis of single-crystal X-ray diffraction data. Compared to the previous structure report, decisive differences are (i) the introduction of a split position for one Li site [occupancy ratio 0.838 (7):0.162 (7)], (ii) the anisotropic refinement of atomic displacement parameters for all atoms, and (iii) a high accuracy of atom positions and unit-cell parameters. The asymmetric unit of Li_13_Si_4_ contains two Si and seven Li atoms. Except for one Li atom situated on a site with symmetry 2/*m*, all other atoms are on mirror planes. The structure consists of isolated Si atoms as well as Si–Si dumbbells surrounded by Li atoms. Each Si atom is either 12- or 13-coordinated. The isolated Si atoms are situated in the *ab* plane at *z* = 0 and are strictly separated from the Si–Si dumbbells at *z* = 0.5.

## Related literature

For details of the structural description of Li_13_Si_4_, see: Frank *et al.* (1975 ▶). For structural data for Li_13_Si_4_ based on computational methods, see: Chevrier *et al.* (2010 ▶). For details of the synthesis, thermodynamic properties and crystal structures of Li_17_Si_4_, Li_16.42_Si_4_ and Li_15_Si_4_, see: Zeilinger & Benson *et al.* (2013 ▶); Zeilinger & Kurylyshyn *et al.* (2013 ▶); Zeilinger & Baran *et al.* (2013 ▶). For further thermodynamic investigations on the Li–Si system, see: Thomas *et al.* (2013 ▶); Wang *et al.* (2013 ▶). The behavior of silicon as anode material upon li­thia­tion/deli­thia­tion is described by Limthongkul *et al.* (2003 ▶) and Obrovac & Christensen (2004 ▶). For *in-situ*/*ex-situ* solid state NMR investigations of structural changes in silicon electrodes for lithium-ion batteries, see: Key *et al.* (2009 ▶, 2011 ▶).

## Experimental

### 

#### Crystal data

Li_13_Si_4_
*M* *_r_* = 202.58
Orthorhombic, 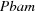

*a* = 7.9488 (4) Å
*b* = 15.1248 (8) Å
*c* = 4.4661 (2) Å
*V* = 536.93 (5) Å^3^
*Z* = 2
Mo *K*α radiation
μ = 0.47 mm^−1^
*T* = 100 K
0.2 × 0.2 × 0.2 mm

#### Data collection

Bruker APEXII CCD diffractometer
Absorption correction: multi-scan (*SADABS*; Bruker, 2008 ▶) *T*_min_ = 0.781, *T*_max_ = 0.818
25938 measured reflections
2429 independent reflections
2333 reflections with *I* > 2σ(*I*)
*R*_int_ = 0.033

#### Refinement

*R*[*F* ^2^ > 2σ(*F* ^2^)] = 0.015
*wR*(*F* ^2^) = 0.044
*S* = 1.08
2429 reflections
60 parameters
Δρ_max_ = 0.68 e Å^−3^
Δρ_min_ = −0.40 e Å^−3^

### 

Data collection: *APEX2* (Bruker, 2008 ▶); cell refinement: *SAINT* (Bruker, 2008 ▶); data reduction: *SAINT*; program(s) used to solve structure: *SHELXS97* (Sheldrick, 2008 ▶); program(s) used to refine structure: *SHELXL97* (Sheldrick, 2008 ▶); molecular graphics: *DIAMOND* (Brandenburg, 2012 ▶); software used to prepare material for publication: *publCIF* (Westrip, 2010 ▶).

## Supplementary Material

Crystal structure: contains datablock(s) global, I. DOI: 10.1107/S1600536813029759/wm2778sup1.cif

Structure factors: contains datablock(s) I. DOI: 10.1107/S1600536813029759/wm2778Isup2.hkl

Additional supplementary materials:  crystallographic information; 3D view; checkCIF report

## Figures and Tables

**Fig. 1.**
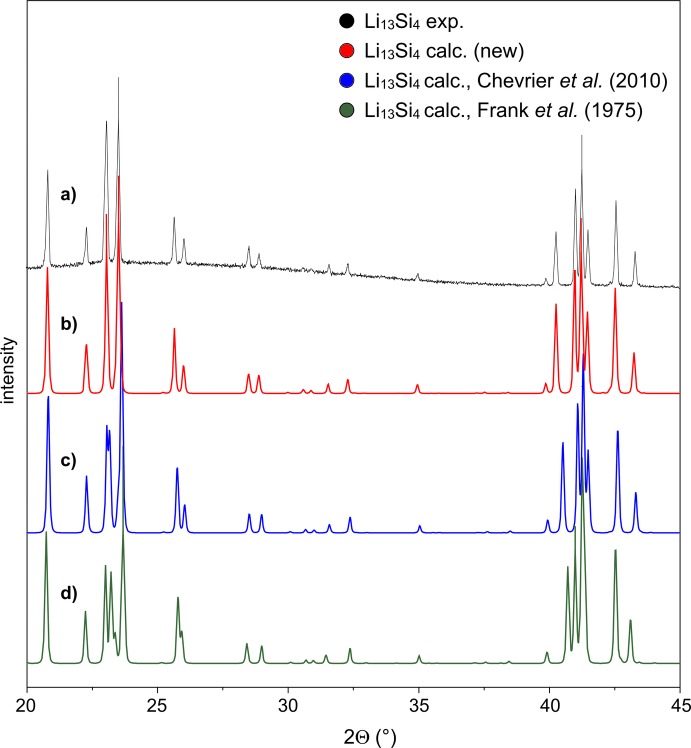
(a) Powder X-ray diffraction pattern of a Li13Si4 sample referenced with the theoretical patterns calculated from (b) single-crystal X-ray data presented herein, previous structural data published by (c) Chevrier et al. (2010) and (d) Frank et al. (1975).

**Fig. 2.**
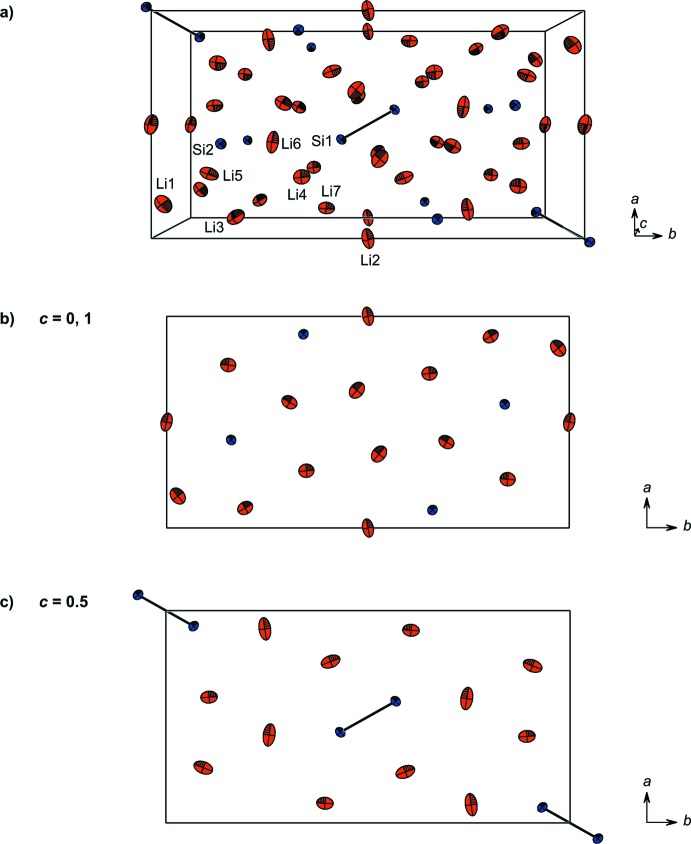
Structure motifs in Li13Si4: (a) perspective view along the crystallographic c-axis of the unit cell of Li13Si4 (atoms of the asymmetric unit are labeled), (b) isolated Si atoms, and (c) Si—Si dumbbells located in different ab-planes (c = 0, 1 and c = 1/2, respectively). Ellipsoids are drawn at a 80% probability level (Li = red, Si = blue; only the major component of Li6 is shown).

## supplementary crystallographic information

### 1. Comment

In the last decade, the demand for high capacity lithium-ion batteries (LIBs) particularly fueled the research on the Li—Si phase system since as anode material, Si theoretically offers a specific capacity of 3579 mA h g^-1^ based on the formation of the metastable phase Li_15_Si_4_ (Obrovac & Christensen, 2004). It is well known that Li_15_Si_4_ is the only Li—Si phase that appears in crystalline form during charging and discharging processes in silicon based LIBs at room temperature (Limthongkul *et al.*, 2003; Obrovac & Christensen, 2004). However, in order to understand the lithiation/delithiation mechanism, X-ray diffraction methods only provide sparse information and therefore other techniques such as *in-situ* / *ex-situ* solid state NMR are frequently used (Key & Bhattacharyya *et al.*, 2009; Key & Morcrette *et al.*, 2011).

Furthermore, a fundamental understanding of the thermodynamic relation of Li—Si phases, especially in the lithium-rich section of the phase diagram, is of considerable importance. Exemplary work is given by Zeilinger & Baran *et al.* (2013), Zeilinger & Benson *et al.* (2013), Zeilinger & Kurylyshyn *et al.* (2013), Thomas *et al.* (2013) and Wang *et al.* (2013). For those studies as well as for NMR investigations it is crucial that all existing Li—Si phases are structurally well characterized since otherwise a phase identification of model compounds can be difficult (Key & Bhattacharyya *et al.*, 2009; Thomas *et al.*, 2013). In the course of our investigations on the thermodynamic properties of Li—Si phases, we identified two new phases, Li_17_Si_4_ (Zeilinger & Benson *et al.*, 2013) and Li_16.42_Si_4_ (Zeilinger & Kurylyshyn *et al.*, 2013). The latter is assigned a high temperature phase existing in a temperature range of 743–891 K, the former decomposes peritectically at 754–759 K. Li_16.42_Si_4_ is compositionally embraced by the lithium-richer phase Li_17_Si_4_ and the lithium-poorer phase Li_13_Si_4_. Since the determination of the Li—Si phase diagram in the aforementioned section is carried out by thermal investigations on various samples with different Li concentrations, the structures of the relevant phases have to be ascertained for an unambiguous assignment of phases in X-ray powder diffraction patterns of those samples. However, the calculated X-ray diffraction pattern of Li_13_Si_4_ based on structural data published by Frank *et al.* (1975) decisively differs from the experimentally observed pattern of a Li_13_Si_4_ sample (Fig. 1). More recent data based on theoretical calculations were reported by Chevrier *et al.* (2010). Yet, the accordingly calculated pattern is slightly but still distinctly different (Fig. 1).

Therefore, we redetermined the structure of Li_13_Si_4_ based on single crystal X-ray diffraction data. As can be seen in Fig. 1, the resulting calculated pattern is in very good agreement with the experimental one. Main differences to the previous single-crystal X-ray structure determination by Frank *et al.* (1975) are i) a significantly more precise determination of atomic positions and unit-cell parameters, respectively (*a* = 7.99 (2) Å, *b* = 15.21 (3) Å, *c* = 4.43 (1) Å compared with *a* = 7.9488 (4) Å, *b* = 15.1248 (8) Å, *c* = 4.4661 (2) Å), ii) an anisotropic refinement of atomic displacement parameters for all atoms and iii) the introduction of a split position for Li6 (occupancy ratio 0.838 (7):0.162 (7)).

Regarding the structure of Li_13_Si_4_ we briefly elaborate on the main structure motifs since this has already been described in detail by Frank *et al.* (1975). Contrary to the lithium-richer phases Li_17_Si_4_, Li_16.42_Si_4_ and Li_15_Si_4_ where all Si atoms are isolated, Li_13_Si_4_ contains Si—Si dumbbells beside isolated Si atoms (Fig. 2a). Li—Si distances range from 2.5173 (2) Å to 3.2283 (7) Å and next nearest neighbor distances are clearly separated, starting off from 4.1899 (2) Å. The shortest Li—Li distance is 2.429 (7) Å and comparable to other Li—Si phases (Zeilinger & Benson *et al.*, 2013). The Si—Si distance within the Si—Si dumbbells is 2.3852 (2) Å, further Si atoms are separated by distances larger than 4.4661 (2) Å. Whereas Si1 is coordinated by 12 Li and one Si atom, Si2 is exclusively surrounded by 12 Li atoms. In addition, dumbbells and isolated Si atoms are strictly separated from each other in a layer like fashion as they are located in different *ab*-planes (Figs. 2 b and c).

### 2. Experimental

In our previous work we reported on thermal investigations by differential scanning calorimetry (DSC) means which were targeting the determination of the lithium-rich section of the Li—Si phase diagram (Zeilinger & Kurylyshyn *et al.* 2013). Various samples with different Li—Si compositions (Li_17_Si_4_, "Li_16.5_Si_4_", "Li_16_Si_4_" and "Li_14_Si_4_") were synthesized. Crystals of Li_13_Si_4_ could be obtained from a sample with a nominal composition "Li_14_Si_4_". For the synthesis of "Li_14_Si_4_" we refer to Zeilinger & Kurylyshyn *et al.* (2013). Li_13_Si_4_ crystals were handled inside an Ar-filled glove box, selected under a microscope and sealed inside 0.3 mm glass capillaries for X-ray diffraction experiments.

### 3. Refinement

For better comparison between the first structure refinement and the current redetermination, atomic coordinates and atom labels were taken from Frank *et al.* (1975). During the structure refinement procedure the positions of two Si and seven Li atoms were confirmed. If refined freely, the site occupation factors of all atoms converged to values very close to full occupancy for the respective sites and were therefore constrained for full occupancy. Extinction was refined to non-significant values and thus excluded from the refinement. Furthermore, an anisotropic refinement of atomic displacement parameters was possible for all atoms. This model resulted in *R*-values of *R*_1_ = 0.020 and *wR*_2_ = 0.059 for all data and residual electron densities of +1.223 e Å^-3^ and -0.740 e Å^-3^. However, the atomic displacement parameters for Li6 on Wyckoff position 4*h* (0.0895 (2), 0.25508 (9), 1/2) indicated a large prolongation in the *x*-direction. Additionally, significant residual electron density (+1.22 e Å^-3^) is located closely to Li6. To account for that, we introduced a split position for Li6. This resulted in markedly better *R*-values of *R*_1_ = 0.016 and *wR*_2_ = 0.044 for all data as well as acceptable residual electron densities of +0.68 e Å^-3^ and -0.40 e Å^-3^. The refined fractions are 0.838 (7) for Li6A and 0.162 (7) for Li6B. An example for a similar introduction of an atom split in lithium-rich Li—Si phases is given by Zeilinger & Kurylyshyn *et al.* (2013).

### Crystal data

**Table:**

Li_13_Si_4_	*D*_x_ = 1.253 Mg m^−^^3^
*M**_r_* = 202.58	Melting point: 995 K
Orthorhombic, *P**b**a**m*	Mo *K*α radiation, λ = 0.71073 Å
Hall symbol: -P 2 2ab	Cell parameters from 9823 reflections
*a* = 7.9488 (4) Å	θ = 2.9–57.3°
*b* = 15.1248 (8) Å	µ = 0.47 mm^−^^1^
*c* = 4.4661 (2) Å	*T* = 100 K
*V* = 536.93 (5) Å^3^	Block, metallic silver
*Z* = 2	0.2 × 0.2 × 0.2 mm
*F*(000) = 190	

### Data collection

**Table:**

Bruker APEXII CCD diffractometer	2429 independent reflections
Radiation source: rotating anode FR591	2333 reflections with *I* > 2σ(*I*)
MONTEL optic monochromator	*R*_int_ = 0.033
Detector resolution: 16 pixels mm^-1^	θ_max_ = 45.3°, θ_min_ = 2.7°
φ– and ω–rotation scans	*h* = −13→15
Absorption correction: multi-scan (*SADABS*; Bruker, 2008)	*k* = −28→30
*T*_min_ = 0.781, *T*_max_ = 0.818	*l* = −8→8
25938 measured reflections	

### Refinement

**Table:**

Refinement on *F*^2^	0 restraints
Least-squares matrix: full	Primary atom site location: structure-invariant direct methods
*R*[*F*^2^ > 2σ(*F*^2^)] = 0.015	Secondary atom site location: difference Fourier map
*wR*(*F*^2^) = 0.044	*w* = 1/[σ^2^(*F*_o_^2^) + (0.0204*P*)^2^ + 0.0373*P*] where *P* = (*F*_o_^2^ + 2*F*_c_^2^)/3
*S* = 1.08	(Δ/σ)_max_ = 0.002
2429 reflections	Δρ_max_ = 0.68 e Å^−^^3^
60 parameters	Δρ_min_ = −0.40 e Å^−^^3^

### Special details

**Table:**

Geometry. All e.s.d.'s (except the e.s.d. in the dihedral angle between two l.s. planes) are estimated using the full covariance matrix. The cell e.s.d.'s are taken into account individually in the estimation of e.s.d.'s in distances, angles and torsion angles; correlations between e.s.d.'s in cell parameters are only used when they are defined by crystal symmetry. An approximate (isotropic) treatment of cell e.s.d.'s is used for estimating e.s.d.'s involving l.s. planes.
Refinement. Refinement of *F*^2^ against all reflections. The weighted *R*-factor *wR* and goodness of fit *S* are based on *F*^2^, conventional *R*-factors *R* are based on *F*, with *F* set to zero for negative *F*^2^. The threshold expression of *F*^2^ > σ(*F*^2^) is used only for calculating *R*-factors(gt) *etc*. and is not relevant to the choice of reflections for refinement. *R*-factors based on *F*^2^ are statistically about twice as large as those based on *F*, and *R*-factors based on all data will be even larger.

### Fractional atomic coordinates and isotropic or equivalent isotropic displacement parameters (Å^2^)

**Table:**

	*x*	*y*	*z*	*U*_iso_*/*U*_eq_	Occ. (<1)
Si1	0.426415 (11)	0.431285 (6)	0.5000	0.00704 (2)	
Si2	0.415184 (11)	0.160356 (6)	0.0000	0.00617 (2)	
Li1	0.15083 (11)	0.02778 (6)	0.0000	0.01635 (13)	
Li2	0.0000	0.5000	0.0000	0.01727 (19)	
Li3	0.09442 (12)	0.19506 (7)	0.0000	0.0242 (2)	
Li4	0.27005 (11)	0.34697 (6)	0.0000	0.01476 (13)	
Li5	0.25998 (11)	0.09271 (6)	0.5000	0.01663 (14)	
Li6A	0.4138 (3)	0.25535 (7)	0.5000	0.0195 (4)	0.838 (7)
Li6B	0.3327 (12)	0.2487 (4)	0.5000	0.0192 (15)	0.162 (7)
Li7	0.09281 (10)	0.39330 (6)	0.5000	0.01429 (13)	

### Atomic displacement parameters (Å^2^)

**Table:**

	*U*^11^	*U*^22^	*U*^33^	*U*^12^	*U*^13^	*U*^23^
Si1	0.00650 (4)	0.00658 (4)	0.00803 (4)	−0.00146 (2)	0.000	0.000
Si2	0.00659 (4)	0.00652 (4)	0.00541 (4)	−0.00044 (2)	0.000	0.000
Li1	0.0192 (3)	0.0173 (3)	0.0126 (3)	−0.0052 (3)	0.000	0.000
Li2	0.0249 (5)	0.0097 (4)	0.0172 (5)	−0.0035 (4)	0.000	0.000
Li3	0.0129 (3)	0.0169 (3)	0.0427 (6)	0.0034 (3)	0.000	0.000
Li4	0.0133 (3)	0.0173 (3)	0.0137 (3)	0.0004 (2)	0.000	0.000
Li5	0.0127 (3)	0.0262 (4)	0.0110 (3)	−0.0061 (3)	0.000	0.000
Li6A	0.0374 (11)	0.0107 (4)	0.0104 (4)	0.0041 (4)	0.000	0.000
Li6B	0.019 (4)	0.017 (2)	0.022 (3)	0.003 (2)	0.000	0.000
Li7	0.0105 (3)	0.0204 (3)	0.0119 (3)	−0.0008 (2)	0.000	0.000

### Geometric parameters (Å, º)

**Table:**

Si1—Si1^i^	2.3852 (2)	Li3—Li6A^xii^	2.7585 (14)
Si1—Li6A	2.6628 (12)	Li3—Li6A^xiii^	2.7585 (14)
Si1—Li5^ii^	2.6762 (8)	Li3—Li5	3.0191 (9)
Si1—Li7	2.7133 (8)	Li3—Li5^viii^	3.0191 (9)
Si1—Li1^iii^	2.7374 (5)	Li3—Li6B	3.038 (7)
Si1—Li1^iv^	2.7374 (5)	Li3—Li6B^viii^	3.038 (7)
Si1—Li5^iii^	2.8559 (9)	Li3—Li6B^xii^	3.168 (7)
Si1—Li4^v^	2.8561 (5)	Li4—Li3^ii^	2.6556 (13)
Si1—Li4	2.8561 (5)	Li4—Li6B	2.728 (3)
Si1—Li6B	2.860 (7)	Li4—Li6B^viii^	2.728 (3)
Si1—Li1^vi^	2.9244 (6)	Li4—Li7	2.7317 (7)
Si1—Li1^ii^	2.9244 (6)	Li4—Li7^viii^	2.7318 (7)
Si2—Li2^vii^	2.5174 (2)	Li4—Li1^iv^	2.8060 (12)
Si2—Li3	2.6031 (9)	Li4—Si2^xiii^	2.8229 (9)
Si2—Li3^ii^	2.6099 (10)	Li4—Si1^viii^	2.8562 (5)
Si2—Li6A	2.6554 (6)	Li4—Li6A^viii^	2.8659 (11)
Si2—Li6A^viii^	2.6554 (6)	Li4—Li6A	2.8659 (11)
Si2—Li6B^viii^	2.684 (3)	Li5—Li6B	2.429 (7)
Si2—Li6B	2.684 (3)	Li5—Li1^v^	2.5892 (6)
Si2—Li5	2.7487 (5)	Li5—Li7^ii^	2.6540 (12)
Si2—Li5^viii^	2.7487 (5)	Li5—Si1^xiii^	2.6762 (8)
Si2—Li7^ii^	2.7638 (5)	Li5—Li6A	2.7472 (18)
Si2—Li7^ix^	2.7638 (5)	Li5—Si2^v^	2.7487 (5)
Si2—Li4^ii^	2.8229 (9)	Li5—Si1^xi^	2.8559 (9)
Li1—Li1^x^	2.5408 (17)	Li5—Li3^v^	3.0191 (9)
Li1—Li3	2.5696 (13)	Li5—Li7^xi^	3.2351 (14)
Li1—Li5^viii^	2.5892 (6)	Li6A—Si2^v^	2.6553 (6)
Li1—Li5	2.5892 (6)	Li6A—Li7^ii^	2.6605 (16)
Li1—Si1^vii^	2.7374 (5)	Li6A—Li3^vi^	2.7585 (14)
Li1—Si1^xi^	2.7374 (5)	Li6A—Li3^ii^	2.7585 (14)
Li1—Li4^vii^	2.8060 (12)	Li6A—Li4^v^	2.8659 (11)
Li1—Li2^vii^	2.8071 (9)	Li6A—Li7	3.296 (2)
Li1—Si1^xii^	2.9244 (6)	Li6A—Li3^v^	3.5021 (19)
Li1—Si1^xiii^	2.9244 (6)	Li6B—Si2^v^	2.684 (3)
Li2—Si2^xiii^	2.5173 (2)	Li6B—Li4^v^	2.728 (3)
Li2—Si2^iv^	2.5174 (2)	Li6B—Li7	2.902 (7)
Li2—Li1^xiii^	2.8071 (9)	Li6B—Li7^ii^	2.981 (7)
Li2—Li1^iv^	2.8071 (9)	Li6B—Li3^v^	3.038 (7)
Li2—Li7^viii^	2.8522 (6)	Li6B—Li3^vi^	3.168 (7)
Li2—Li7^xiv^	2.8522 (6)	Li6B—Li3^ii^	3.168 (7)
Li2—Li7^xv^	2.8522 (6)	Li7—Li5^xiii^	2.6540 (12)
Li2—Li7	2.8522 (6)	Li7—Li6A^xiii^	2.6605 (16)
Li2—Li4^xv^	3.1567 (9)	Li7—Li4^v^	2.7317 (7)
Li2—Li4	3.1567 (9)	Li7—Si2^xiii^	2.7638 (5)
Li2—Li5^iii^	3.2546 (7)	Li7—Si2^xvi^	2.7638 (5)
Li2—Li5^iv^	3.2546 (7)	Li7—Li2^v^	2.8522 (6)
Li3—Si2^xiii^	2.6099 (10)	Li7—Li6B^xiii^	2.981 (7)
Li3—Li4^xiii^	2.6556 (13)	Li7—Li5^iii^	3.2351 (14)
Li3—Li4	2.6884 (13)		
			
Si1^i^—Si1—Li6A	152.78 (5)	Li3^ii^—Li4—Li7	124.20 (2)
Si1^i^—Si1—Li5^ii^	68.43 (2)	Li3—Li4—Li7	87.21 (3)
Li6A—Si1—Li5^ii^	84.35 (5)	Li6B—Li4—Li7	64.21 (14)
Si1^i^—Si1—Li7	131.60 (2)	Li6B^viii^—Li4—Li7	154.5 (2)
Li6A—Si1—Li7	75.63 (5)	Li3^ii^—Li4—Li7^viii^	124.20 (2)
Li5^ii^—Si1—Li7	159.98 (3)	Li3—Li4—Li7^viii^	87.21 (3)
Si1^i^—Si1—Li1^iii^	69.231 (19)	Li6B—Li4—Li7^viii^	154.5 (2)
Li6A—Si1—Li1^iii^	121.62 (2)	Li6B^viii^—Li4—Li7^viii^	64.21 (14)
Li5^ii^—Si1—Li1^iii^	107.13 (2)	Li7—Li4—Li7^viii^	109.66 (4)
Li7—Si1—Li1^iii^	83.90 (2)	Li3^ii^—Li4—Li1^iv^	90.90 (4)
Si1^i^—Si1—Li1^iv^	69.231 (19)	Li3—Li4—Li1^iv^	161.67 (4)
Li6A—Si1—Li1^iv^	121.62 (2)	Li6B—Li4—Li1^iv^	119.34 (14)
Li5^ii^—Si1—Li1^iv^	107.13 (2)	Li6B^viii^—Li4—Li1^iv^	119.34 (14)
Li7—Si1—Li1^iv^	83.90 (2)	Li7—Li4—Li1^iv^	82.28 (3)
Li1^iii^—Si1—Li1^iv^	109.32 (3)	Li7^viii^—Li4—Li1^iv^	82.28 (3)
Si1^i^—Si1—Li5^iii^	60.62 (2)	Li3^ii^—Li4—Si2^xiii^	163.90 (4)
Li6A—Si1—Li5^iii^	146.60 (5)	Li3—Li4—Si2^xiii^	56.47 (3)
Li5^ii^—Si1—Li5^iii^	129.047 (16)	Li6B—Li4—Si2^xiii^	99.3 (2)
Li7—Si1—Li5^iii^	70.97 (3)	Li6B^viii^—Li4—Si2^xiii^	99.3 (2)
Li1^iii^—Si1—Li5^iii^	55.100 (14)	Li7—Li4—Si2^xiii^	59.65 (2)
Li1^iv^—Si1—Li5^iii^	55.100 (14)	Li7^viii^—Li4—Si2^xiii^	59.65 (2)
Si1^i^—Si1—Li4^v^	127.050 (14)	Li1^iv^—Li4—Si2^xiii^	105.20 (3)
Li6A—Si1—Li4^v^	62.45 (3)	Li3^ii^—Li4—Si1	71.60 (2)
Li5^ii^—Si1—Li4^v^	111.752 (19)	Li3—Li4—Si1	127.412 (15)
Li7—Si1—Li4^v^	58.678 (17)	Li6B—Li4—Si1	61.57 (14)
Li1^iii^—Si1—Li4^v^	60.17 (2)	Li6B^viii^—Li4—Si1	143.5 (2)
Li1^iv^—Si1—Li4^v^	141.10 (3)	Li7—Li4—Si1	58.048 (18)
Li5^iii^—Si1—Li4^v^	98.97 (2)	Li7^viii^—Li4—Si1	138.51 (4)
Si1^i^—Si1—Li4	127.051 (14)	Li1^iv^—Li4—Si1	57.815 (16)
Li6A—Si1—Li4	62.45 (3)	Si2^xiii^—Li4—Si1	116.896 (19)
Li5^ii^—Si1—Li4	111.752 (19)	Li3^ii^—Li4—Si1^viii^	71.60 (2)
Li7—Si1—Li4	58.678 (17)	Li3—Li4—Si1^viii^	127.413 (15)
Li1^iii^—Si1—Li4	141.10 (3)	Li6B—Li4—Si1^viii^	143.5 (2)
Li1^iv^—Si1—Li4	60.17 (2)	Li6B^viii^—Li4—Si1^viii^	61.57 (14)
Li5^iii^—Si1—Li4	98.97 (2)	Li7—Li4—Si1^viii^	138.51 (4)
Li4^v^—Si1—Li4	102.86 (3)	Li7^viii^—Li4—Si1^viii^	58.048 (18)
Si1^i^—Si1—Li6B	165.72 (19)	Li1^iv^—Li4—Si1^viii^	57.814 (16)
Li6A—Si1—Li6B	12.94 (15)	Si2^xiii^—Li4—Si1^viii^	116.896 (19)
Li5^ii^—Si1—Li6B	97.30 (19)	Si1—Li4—Si1^viii^	102.86 (3)
Li7—Si1—Li6B	62.68 (19)	Li3^ii^—Li4—Li6A^viii^	59.80 (4)
Li1^iii^—Si1—Li6B	117.15 (8)	Li3—Li4—Li6A^viii^	78.11 (4)
Li1^iv^—Si1—Li6B	117.15 (8)	Li6B—Li4—Li6A^viii^	107.55 (12)
Li5^iii^—Si1—Li6B	133.66 (19)	Li6B^viii^—Li4—Li6A^viii^	13.10 (18)
Li4^v^—Si1—Li6B	57.02 (7)	Li7—Li4—Li6A^viii^	165.17 (5)
Li4—Si1—Li6B	57.02 (7)	Li7^viii^—Li4—Li6A^viii^	72.11 (3)
Si1^i^—Si1—Li1^vi^	61.073 (16)	Li1^iv^—Li4—Li6A^viii^	112.45 (4)
Li6A—Si1—Li1^vi^	103.56 (3)	Si2^xiii^—Li4—Li6A^viii^	112.31 (5)
Li5^ii^—Si1—Li1^vi^	54.856 (16)	Si1—Li4—Li6A^viii^	130.66 (5)
Li7—Si1—Li1^vi^	129.866 (14)	Si1^viii^—Li4—Li6A^viii^	55.47 (3)
Li1^iii^—Si1—Li1^vi^	53.20 (3)	Li3^ii^—Li4—Li6A	59.80 (4)
Li1^iv^—Si1—Li1^vi^	130.304 (9)	Li3—Li4—Li6A	78.10 (4)
Li5^iii^—Si1—Li1^vi^	97.78 (2)	Li6B—Li4—Li6A	13.10 (18)
Li4^v^—Si1—Li1^vi^	76.29 (2)	Li6B^viii^—Li4—Li6A	107.55 (12)
Li4—Si1—Li1^vi^	163.14 (2)	Li7—Li4—Li6A	72.11 (3)
Li6B—Si1—Li1^vi^	111.30 (11)	Li7^viii^—Li4—Li6A	165.17 (5)
Si1^i^—Si1—Li1^ii^	61.073 (16)	Li1^iv^—Li4—Li6A	112.45 (4)
Li6A—Si1—Li1^ii^	103.56 (3)	Si2^xiii^—Li4—Li6A	112.31 (5)
Li5^ii^—Si1—Li1^ii^	54.856 (16)	Si1—Li4—Li6A	55.47 (3)
Li7—Si1—Li1^ii^	129.866 (14)	Si1^viii^—Li4—Li6A	130.66 (5)
Li1^iii^—Si1—Li1^ii^	130.304 (9)	Li6A^viii^—Li4—Li6A	102.37 (5)
Li1^iv^—Si1—Li1^ii^	53.20 (3)	Li6B—Li5—Li1	116.63 (6)
Li5^iii^—Si1—Li1^ii^	97.78 (2)	Li6B—Li5—Li1^v^	116.63 (6)
Li4^v^—Si1—Li1^ii^	163.14 (2)	Li1—Li5—Li1^v^	119.19 (5)
Li4—Si1—Li1^ii^	76.29 (2)	Li6B—Li5—Li7^ii^	71.7 (2)
Li6B—Si1—Li1^ii^	111.30 (11)	Li1—Li5—Li7^ii^	111.36 (3)
Li1^vi^—Si1—Li1^ii^	99.56 (3)	Li1^v^—Li5—Li7^ii^	111.36 (3)
Li2^vii^—Si2—Li3	117.17 (2)	Li6B—Li5—Si1^xiii^	111.6 (2)
Li2^vii^—Si2—Li3^ii^	131.38 (2)	Li1—Li5—Si1^xiii^	67.45 (3)
Li3—Si2—Li3^ii^	111.451 (16)	Li1^v^—Li5—Si1^xiii^	67.45 (3)
Li2^vii^—Si2—Li6A	121.49 (2)	Li7^ii^—Li5—Si1^xiii^	176.78 (5)
Li3—Si2—Li6A	83.51 (5)	Li6B—Li5—Li6A	12.67 (19)
Li3^ii^—Si2—Li6A	63.18 (4)	Li1—Li5—Li6A	119.26 (3)
Li2^vii^—Si2—Li6A^viii^	121.49 (2)	Li1^v^—Li5—Li6A	119.26 (3)
Li3—Si2—Li6A^viii^	83.51 (5)	Li7^ii^—Li5—Li6A	58.99 (5)
Li3^ii^—Si2—Li6A^viii^	63.18 (4)	Si1^xiii^—Li5—Li6A	124.23 (6)
Li6A—Si2—Li6A^viii^	114.48 (4)	Li6B—Li5—Si2	62.07 (9)
Li2^vii^—Si2—Li6B^viii^	123.03 (10)	Li1—Li5—Si2	65.853 (16)
Li3—Si2—Li6B^viii^	70.1 (2)	Li1^v^—Li5—Si2	172.86 (4)
Li3^ii^—Si2—Li6B^viii^	73.51 (18)	Li7^ii^—Li5—Si2	61.506 (17)
Li6A—Si2—Li6B^viii^	115.43 (10)	Si1^xiii^—Li5—Si2	119.680 (19)
Li6A^viii^—Si2—Li6B^viii^	14.03 (17)	Li6A—Li5—Si2	57.78 (2)
Li2^vii^—Si2—Li6B	123.03 (10)	Li6B—Li5—Si2^v^	62.07 (9)
Li3—Si2—Li6B	70.1 (2)	Li1—Li5—Si2^v^	172.86 (4)
Li3^ii^—Si2—Li6B	73.51 (18)	Li1^v^—Li5—Si2^v^	65.854 (16)
Li6A—Si2—Li6B	14.03 (17)	Li7^ii^—Li5—Si2^v^	61.506 (17)
Li6A^viii^—Si2—Li6B	115.43 (10)	Si1^xiii^—Li5—Si2^v^	119.680 (19)
Li6B^viii^—Si2—Li6B	112.6 (2)	Li6A—Li5—Si2^v^	57.78 (2)
Li2^vii^—Si2—Li5	76.21 (2)	Si2—Li5—Si2^v^	108.66 (3)
Li3—Si2—Li5	68.62 (2)	Li6B—Li5—Si1^xi^	162.5 (2)
Li3^ii^—Si2—Li5	123.840 (16)	Li1—Li5—Si1^xi^	60.12 (2)
Li6A—Si2—Li5	61.08 (4)	Li1^v^—Li5—Si1^xi^	60.12 (2)
Li6A^viii^—Si2—Li5	151.98 (5)	Li7^ii^—Li5—Si1^xi^	125.82 (4)
Li6B^viii^—Si2—Li5	138.7 (2)	Si1^xiii^—Li5—Si1^xi^	50.954 (16)
Li6B—Si2—Li5	53.11 (15)	Li6A—Li5—Si1^xi^	175.18 (6)
Li2^vii^—Si2—Li5^viii^	76.21 (2)	Si2—Li5—Si1^xi^	123.439 (18)
Li3—Si2—Li5^viii^	68.62 (2)	Si2^v^—Li5—Si1^xi^	123.440 (18)
Li3^ii^—Si2—Li5^viii^	123.840 (16)	Li6B—Li5—Li3	66.78 (13)
Li6A—Si2—Li5^viii^	151.98 (5)	Li1—Li5—Li3	53.88 (3)
Li6A^viii^—Si2—Li5^viii^	61.08 (4)	Li1^v^—Li5—Li3	133.34 (4)
Li6B^viii^—Si2—Li5^viii^	53.11 (15)	Li7^ii^—Li5—Li3	113.18 (3)
Li6B—Si2—Li5^viii^	138.7 (2)	Si1^xiii^—Li5—Li3	68.76 (2)
Li5—Si2—Li5^viii^	108.66 (3)	Li6A—Li5—Li3	74.63 (4)
Li2^vii^—Si2—Li7^ii^	65.184 (19)	Si2—Li5—Li3	53.408 (19)
Li3—Si2—Li7^ii^	124.026 (15)	Si2^v^—Li5—Li3	127.28 (4)
Li3^ii^—Si2—Li7^ii^	88.12 (3)	Si1^xi^—Li5—Li3	102.25 (3)
Li6A—Si2—Li7^ii^	58.76 (3)	Li6B—Li5—Li3^v^	66.78 (13)
Li6A^viii^—Si2—Li7^ii^	147.18 (5)	Li1—Li5—Li3^v^	133.34 (4)
Li6B^viii^—Si2—Li7^ii^	160.6 (2)	Li1^v^—Li5—Li3^v^	53.88 (3)
Li6B—Si2—Li7^ii^	66.34 (14)	Li7^ii^—Li5—Li3^v^	113.18 (3)
Li5—Si2—Li7^ii^	57.56 (2)	Si1^xiii^—Li5—Li3^v^	68.76 (2)
Li5^viii^—Si2—Li7^ii^	140.93 (3)	Li6A—Li5—Li3^v^	74.63 (4)
Li2^vii^—Si2—Li7^ix^	65.184 (18)	Si2—Li5—Li3^v^	127.28 (4)
Li3—Si2—Li7^ix^	124.026 (15)	Si2^v^—Li5—Li3^v^	53.408 (19)
Li3^ii^—Si2—Li7^ix^	88.12 (3)	Si1^xi^—Li5—Li3^v^	102.25 (3)
Li6A—Si2—Li7^ix^	147.18 (5)	Li3—Li5—Li3^v^	95.40 (4)
Li6A^viii^—Si2—Li7^ix^	58.77 (3)	Li6B—Li5—Li7^xi^	145.0 (2)
Li6B^viii^—Si2—Li7^ix^	66.34 (14)	Li1—Li5—Li7^xi^	76.56 (3)
Li6B—Si2—Li7^ix^	160.6 (2)	Li1^v^—Li5—Li7^xi^	76.56 (3)
Li5—Si2—Li7^ix^	140.93 (3)	Li7^ii^—Li5—Li7^xi^	73.37 (4)
Li5^viii^—Si2—Li7^ix^	57.56 (2)	Si1^xiii^—Li5—Li7^xi^	103.41 (3)
Li7^ii^—Si2—Li7^ix^	107.80 (3)	Li6A—Li5—Li7^xi^	132.36 (5)
Li2^vii^—Si2—Li4^ii^	72.216 (17)	Si2—Li5—Li7^xi^	100.64 (2)
Li3—Si2—Li4^ii^	170.62 (3)	Si2^v^—Li5—Li7^xi^	100.64 (2)
Li3^ii^—Si2—Li4^ii^	59.16 (3)	Si1^xi^—Li5—Li7^xi^	52.46 (2)
Li6A—Si2—Li4^ii^	91.45 (5)	Li3—Li5—Li7^xi^	129.47 (2)
Li6A^viii^—Si2—Li4^ii^	91.45 (5)	Li3^v^—Li5—Li7^xi^	129.47 (2)
Li6B^viii^—Si2—Li4^ii^	105.3 (2)	Si2^v^—Li6A—Si2	114.48 (4)
Li6B—Si2—Li4^ii^	105.3 (2)	Si2^v^—Li6A—Li7^ii^	62.65 (2)
Li5—Si2—Li4^ii^	115.713 (19)	Si2—Li6A—Li7^ii^	62.65 (2)
Li5^viii^—Si2—Li4^ii^	115.713 (19)	Si2^v^—Li6A—Si1	122.72 (2)
Li7^ii^—Si2—Li4^ii^	58.534 (15)	Si2—Li6A—Si1	122.72 (2)
Li7^ix^—Si2—Li4^ii^	58.534 (15)	Li7^ii^—Li6A—Si1	145.53 (10)
Li1^x^—Li1—Li3	99.26 (5)	Si2^v^—Li6A—Li5	61.14 (3)
Li1^x^—Li1—Li5^viii^	116.21 (3)	Si2—Li6A—Li5	61.14 (3)
Li3—Li1—Li5^viii^	71.64 (3)	Li7^ii^—Li6A—Li5	58.76 (3)
Li1^x^—Li1—Li5	116.21 (3)	Si1—Li6A—Li5	155.71 (9)
Li3—Li1—Li5	71.64 (3)	Si2^v^—Li6A—Li3^vi^	57.60 (2)
Li5^viii^—Li1—Li5	119.19 (5)	Si2—Li6A—Li3^vi^	145.67 (8)
Li1^x^—Li1—Si1^vii^	67.17 (2)	Li7^ii^—Li6A—Li3^vi^	87.22 (5)
Li3—Li1—Si1^vii^	119.07 (2)	Si1—Li6A—Li3^vi^	73.07 (4)
Li5^viii^—Li1—Si1^vii^	64.776 (18)	Li5—Li6A—Li3^vi^	118.37 (3)
Li5—Li1—Si1^vii^	168.80 (4)	Si2^v^—Li6A—Li3^ii^	145.67 (8)
Li1^x^—Li1—Si1^xi^	67.17 (2)	Si2—Li6A—Li3^ii^	57.61 (2)
Li3—Li1—Si1^xi^	119.07 (2)	Li7^ii^—Li6A—Li3^ii^	87.22 (5)
Li5^viii^—Li1—Si1^xi^	168.80 (4)	Si1—Li6A—Li3^ii^	73.07 (4)
Li5—Li1—Si1^xi^	64.776 (18)	Li5—Li6A—Li3^ii^	118.37 (3)
Si1^vii^—Li1—Si1^xi^	109.32 (3)	Li3^vi^—Li6A—Li3^ii^	108.10 (8)
Li1^x^—Li1—Li4^vii^	83.64 (4)	Si2^v^—Li6A—Li4	156.71 (9)
Li3—Li1—Li4^vii^	177.10 (5)	Si2—Li6A—Li4	66.92 (2)
Li5^viii^—Li1—Li4^vii^	107.13 (3)	Li7^ii^—Li6A—Li4	128.45 (2)
Li5—Li1—Li4^vii^	107.13 (3)	Si1—Li6A—Li4	62.08 (3)
Si1^vii^—Li1—Li4^vii^	62.011 (17)	Li5—Li6A—Li4	104.80 (6)
Si1^xi^—Li1—Li4^vii^	62.012 (18)	Li3^vi^—Li6A—Li4	134.98 (5)
Li1^x^—Li1—Li2^vii^	152.08 (6)	Li3^ii^—Li6A—Li4	56.31 (3)
Li3—Li1—Li2^vii^	108.66 (4)	Si2^v^—Li6A—Li4^v^	66.92 (2)
Li5^viii^—Li1—Li2^vii^	74.07 (3)	Si2—Li6A—Li4^v^	156.71 (9)
Li5—Li1—Li2^vii^	74.07 (3)	Li7^ii^—Li6A—Li4^v^	128.45 (2)
Si1^vii^—Li1—Li2^vii^	98.16 (2)	Si1—Li6A—Li4^v^	62.08 (3)
Si1^xi^—Li1—Li2^vii^	98.16 (2)	Li5—Li6A—Li4^v^	104.80 (6)
Li4^vii^—Li1—Li2^vii^	68.44 (3)	Li3^vi^—Li6A—Li4^v^	56.31 (3)
Li1^x^—Li1—Si2	155.65 (6)	Li3^ii^—Li6A—Li4^v^	134.98 (5)
Li3—Li1—Si2	56.39 (3)	Li4—Li6A—Li4^v^	102.37 (5)
Li5^viii^—Li1—Si2	59.72 (2)	Si2^v^—Li6A—Li7	110.22 (5)
Li5—Li1—Si2	59.72 (2)	Si2—Li6A—Li7	110.22 (5)
Si1^vii^—Li1—Si2	122.029 (17)	Li7^ii^—Li6A—Li7	161.59 (8)
Si1^xi^—Li1—Si2	122.029 (17)	Si1—Li6A—Li7	52.88 (3)
Li4^vii^—Li1—Si2	120.71 (4)	Li5—Li6A—Li7	102.83 (7)
Li2^vii^—Li1—Si2	52.269 (16)	Li3^vi^—Li6A—Li7	103.34 (4)
Li1^x^—Li1—Si1^xii^	59.63 (2)	Li3^ii^—Li6A—Li7	103.34 (4)
Li3—Li1—Si1^xii^	71.64 (2)	Li4—Li6A—Li7	52.06 (3)
Li5^viii^—Li1—Si1^xii^	57.691 (19)	Li4^v^—Li6A—Li7	52.06 (3)
Li5—Li1—Si1^xii^	141.50 (4)	Si2^v^—Li6A—Li3	113.47 (6)
Si1^vii^—Li1—Si1^xii^	49.695 (9)	Si2—Li6A—Li3	47.61 (3)
Si1^xi^—Li1—Si1^xii^	126.80 (3)	Li7^ii^—Li6A—Li3	99.65 (4)
Li4^vii^—Li1—Si1^xii^	110.06 (2)	Si1—Li6A—Li3	106.70 (5)
Li2^vii^—Li1—Si1^xii^	129.406 (14)	Li5—Li6A—Li3	56.23 (4)
Si2—Li1—Si1^xii^	107.16 (2)	Li3^vi^—Li6A—Li3	164.63 (7)
Li1^x^—Li1—Si1^xiii^	59.63 (2)	Li3^ii^—Li6A—Li3	86.09 (2)
Li3—Li1—Si1^xiii^	71.64 (2)	Li4—Li6A—Li3	48.69 (3)
Li5^viii^—Li1—Si1^xiii^	141.50 (4)	Li4^v^—Li6A—Li3	109.49 (7)
Li5—Li1—Si1^xiii^	57.691 (19)	Li7—Li6A—Li3	66.64 (4)
Si1^vii^—Li1—Si1^xiii^	126.80 (3)	Si2^v^—Li6A—Li3^v^	47.61 (3)
Si1^xi^—Li1—Si1^xiii^	49.695 (9)	Si2—Li6A—Li3^v^	113.47 (6)
Li4^vii^—Li1—Si1^xiii^	110.06 (2)	Li7^ii^—Li6A—Li3^v^	99.65 (4)
Li2^vii^—Li1—Si1^xiii^	129.406 (14)	Si1—Li6A—Li3^v^	106.70 (5)
Si2—Li1—Si1^xiii^	107.16 (2)	Li5—Li6A—Li3^v^	56.23 (4)
Si1^xii^—Li1—Si1^xiii^	99.56 (3)	Li3^vi^—Li6A—Li3^v^	86.09 (2)
Si2^xiii^—Li2—Si2^iv^	180.000 (4)	Li3^ii^—Li6A—Li3^v^	164.63 (7)
Si2^xiii^—Li2—Li1^xiii^	65.859 (17)	Li4—Li6A—Li3^v^	109.48 (7)
Si2^iv^—Li2—Li1^xiii^	114.141 (17)	Li4^v^—Li6A—Li3^v^	48.69 (3)
Si2^xiii^—Li2—Li1^iv^	114.142 (17)	Li7—Li6A—Li3^v^	66.64 (4)
Si2^iv^—Li2—Li1^iv^	65.859 (17)	Li3—Li6A—Li3^v^	79.23 (5)
Li1^xiii^—Li2—Li1^iv^	180.0	Li5—Li6B—Si2^v^	64.82 (11)
Si2^xiii^—Li2—Li7^viii^	61.584 (16)	Li5—Li6B—Si2	64.82 (11)
Si2^iv^—Li2—Li7^viii^	118.416 (16)	Si2^v^—Li6B—Si2	112.6 (2)
Li1^xiii^—Li2—Li7^viii^	99.849 (19)	Li5—Li6B—Li4	119.1 (2)
Li1^iv^—Li2—Li7^viii^	80.151 (19)	Si2^v^—Li6B—Li4	175.5 (4)
Si2^xiii^—Li2—Li7^xiv^	118.417 (16)	Si2—Li6B—Li4	68.579 (18)
Si2^iv^—Li2—Li7^xiv^	61.583 (16)	Li5—Li6B—Li4^v^	119.1 (2)
Li1^xiii^—Li2—Li7^xiv^	80.151 (19)	Si2^v^—Li6B—Li4^v^	68.579 (19)
Li1^iv^—Li2—Li7^xiv^	99.849 (19)	Si2—Li6B—Li4^v^	175.5 (4)
Li7^viii^—Li2—Li7^xiv^	180.0	Li4—Li6B—Li4^v^	109.86 (19)
Si2^xiii^—Li2—Li7^xv^	118.417 (16)	Li5—Li6B—Si1	178.7 (4)
Si2^iv^—Li2—Li7^xv^	61.583 (16)	Si2^v^—Li6B—Si1	114.7 (2)
Li1^xiii^—Li2—Li7^xv^	80.151 (19)	Si2—Li6B—Si1	114.7 (2)
Li1^iv^—Li2—Li7^xv^	99.849 (19)	Li4—Li6B—Si1	61.41 (11)
Li7^viii^—Li2—Li7^xv^	76.94 (3)	Li4^v^—Li6B—Si1	61.41 (11)
Li7^xiv^—Li2—Li7^xv^	103.06 (3)	Li5—Li6B—Li7	125.1 (4)
Si2^xiii^—Li2—Li7	61.584 (16)	Si2^v^—Li6B—Li7	122.40 (15)
Si2^iv^—Li2—Li7	118.417 (16)	Si2—Li6B—Li7	122.40 (15)
Li1^xiii^—Li2—Li7	99.850 (19)	Li4—Li6B—Li7	57.95 (11)
Li1^iv^—Li2—Li7	80.151 (19)	Li4^v^—Li6B—Li7	57.95 (11)
Li7^viii^—Li2—Li7	103.06 (3)	Si1—Li6B—Li7	56.18 (10)
Li7^xiv^—Li2—Li7	76.94 (3)	Li5—Li6B—Li7^ii^	57.68 (13)
Li7^xv^—Li2—Li7	180.0	Si2^v^—Li6B—Li7^ii^	58.12 (12)
Si2^xiii^—Li2—Li4^xv^	121.622 (16)	Si2—Li6B—Li7^ii^	58.12 (12)
Si2^iv^—Li2—Li4^xv^	58.378 (16)	Li4—Li6B—Li7^ii^	121.27 (18)
Li1^xiii^—Li2—Li4^xv^	55.76 (2)	Li4^v^—Li6B—Li7^ii^	121.27 (18)
Li1^iv^—Li2—Li4^xv^	124.24 (2)	Si1—Li6B—Li7^ii^	121.0 (3)
Li7^viii^—Li2—Li4^xv^	126.209 (14)	Li7—Li6B—Li7^ii^	177.2 (4)
Li7^xiv^—Li2—Li4^xv^	53.791 (15)	Li5—Li6B—Li3	65.94 (17)
Li7^xv^—Li2—Li4^xv^	53.791 (15)	Si2^v^—Li6B—Li3	129.1 (3)
Li7—Li2—Li4^xv^	126.210 (14)	Si2—Li6B—Li3	53.69 (8)
Si2^xiii^—Li2—Li4	58.378 (16)	Li4—Li6B—Li3	55.26 (9)
Si2^iv^—Li2—Li4	121.622 (16)	Li4^v^—Li6B—Li3	129.3 (3)
Li1^xiii^—Li2—Li4	124.24 (2)	Si1—Li6B—Li3	114.84 (14)
Li1^iv^—Li2—Li4	55.76 (2)	Li7—Li6B—Li3	77.97 (19)
Li7^viii^—Li2—Li4	53.791 (14)	Li7^ii^—Li6B—Li3	103.89 (14)
Li7^xiv^—Li2—Li4	126.210 (14)	Li5—Li6B—Li3^v^	65.94 (17)
Li7^xv^—Li2—Li4	126.210 (14)	Si2^v^—Li6B—Li3^v^	53.69 (8)
Li7—Li2—Li4	53.790 (15)	Si2—Li6B—Li3^v^	129.1 (3)
Li4^xv^—Li2—Li4	180.0	Li4—Li6B—Li3^v^	129.3 (3)
Si2^xiii^—Li2—Li5^iii^	124.896 (15)	Li4^v^—Li6B—Li3^v^	55.26 (9)
Si2^iv^—Li2—Li5^iii^	55.104 (15)	Si1—Li6B—Li3^v^	114.84 (14)
Li1^xiii^—Li2—Li5^iii^	130.096 (14)	Li7—Li6B—Li3^v^	77.97 (19)
Li1^iv^—Li2—Li5^iii^	49.904 (14)	Li7^ii^—Li6B—Li3^v^	103.89 (14)
Li7^viii^—Li2—Li5^iii^	129.00 (2)	Li3—Li6B—Li3^v^	94.6 (3)
Li7^xiv^—Li2—Li5^iii^	51.00 (2)	Li5—Li6B—Li3^vi^	114.65 (17)
Li7^xv^—Li2—Li5^iii^	116.42 (2)	Si2^v^—Li6B—Li3^vi^	52.18 (10)
Li7—Li2—Li5^iii^	63.58 (2)	Si2—Li6B—Li3^vi^	124.0 (3)
Li4^xv^—Li2—Li5^iii^	94.745 (17)	Li4—Li6B—Li3^vi^	123.4 (3)
Li4—Li2—Li5^iii^	85.255 (17)	Li4^v^—Li6B—Li3^vi^	52.90 (9)
Si2^xiii^—Li2—Li5^iv^	124.896 (15)	Si1—Li6B—Li3^vi^	64.52 (16)
Si2^iv^—Li2—Li5^iv^	55.104 (15)	Li7—Li6B—Li3^vi^	103.25 (14)
Li1^xiii^—Li2—Li5^iv^	130.096 (14)	Li7^ii^—Li6B—Li3^vi^	74.81 (18)
Li1^iv^—Li2—Li5^iv^	49.904 (14)	Li3—Li6B—Li3^vi^	177.4 (3)
Li7^viii^—Li2—Li5^iv^	63.58 (2)	Li3^v^—Li6B—Li3^vi^	87.879 (16)
Li7^xiv^—Li2—Li5^iv^	116.42 (2)	Li5—Li6B—Li3^ii^	114.65 (17)
Li7^xv^—Li2—Li5^iv^	51.00 (2)	Si2^v^—Li6B—Li3^ii^	124.0 (3)
Li7—Li2—Li5^iv^	129.00 (2)	Si2—Li6B—Li3^ii^	52.18 (10)
Li4^xv^—Li2—Li5^iv^	94.745 (17)	Li4—Li6B—Li3^ii^	52.90 (9)
Li4—Li2—Li5^iv^	85.255 (17)	Li4^v^—Li6B—Li3^ii^	123.4 (3)
Li5^iii^—Li2—Li5^iv^	86.65 (2)	Si1—Li6B—Li3^ii^	64.52 (16)
Li1—Li3—Si2	68.32 (3)	Li7—Li6B—Li3^ii^	103.25 (14)
Li1—Li3—Si2^xiii^	156.96 (5)	Li7^ii^—Li6B—Li3^ii^	74.81 (18)
Si2—Li3—Si2^xiii^	134.72 (4)	Li3—Li6B—Li3^ii^	87.879 (16)
Li1—Li3—Li4^xiii^	86.20 (4)	Li3^v^—Li6B—Li3^ii^	177.4 (3)
Si2—Li3—Li4^xiii^	154.51 (5)	Li3^vi^—Li6B—Li3^ii^	89.6 (3)
Si2^xiii^—Li3—Li4^xiii^	70.77 (3)	Li5^xiii^—Li7—Li6A^xiii^	62.25 (5)
Li1—Li3—Li4	138.67 (5)	Li5^xiii^—Li7—Si1	163.20 (4)
Si2—Li3—Li4	70.35 (3)	Li6A^xiii^—Li7—Si1	134.55 (6)
Si2^xiii^—Li3—Li4	64.37 (3)	Li5^xiii^—Li7—Li4^v^	122.31 (2)
Li4^xiii^—Li3—Li4	135.14 (4)	Li6A^xiii^—Li7—Li4^v^	93.38 (4)
Li1—Li3—Li6A^xii^	111.01 (3)	Si1—Li7—Li4^v^	63.27 (2)
Si2—Li3—Li6A^xii^	124.37 (4)	Li5^xiii^—Li7—Li4	122.31 (2)
Si2^xiii^—Li3—Li6A^xii^	59.21 (3)	Li6A^xiii^—Li7—Li4	93.38 (4)
Li4^xiii^—Li3—Li6A^xii^	63.89 (4)	Si1—Li7—Li4	63.27 (2)
Li4—Li3—Li6A^xii^	92.17 (4)	Li4^v^—Li7—Li4	109.66 (4)
Li1—Li3—Li6A^xiii^	111.01 (3)	Li5^xiii^—Li7—Si2^xiii^	60.933 (17)
Si2—Li3—Li6A^xiii^	124.37 (4)	Li6A^xiii^—Li7—Si2^xiii^	58.58 (2)
Si2^xiii^—Li3—Li6A^xiii^	59.21 (3)	Si1—Li7—Si2^xiii^	124.156 (15)
Li4^xiii^—Li3—Li6A^xiii^	63.89 (4)	Li4^v^—Li7—Si2^xiii^	148.06 (4)
Li4—Li3—Li6A^xiii^	92.17 (4)	Li4—Li7—Si2^xiii^	61.815 (18)
Li6A^xii^—Li3—Li6A^xiii^	108.10 (8)	Li5^xiii^—Li7—Si2^xvi^	60.933 (17)
Li1—Li3—Li5	54.48 (2)	Li6A^xiii^—Li7—Si2^xvi^	58.58 (2)
Si2—Li3—Li5	57.97 (2)	Si1—Li7—Si2^xvi^	124.156 (15)
Si2^xiii^—Li3—Li5	131.88 (2)	Li4^v^—Li7—Si2^xvi^	61.815 (18)
Li4^xiii^—Li3—Li5	107.49 (3)	Li4—Li7—Si2^xvi^	148.06 (4)
Li4—Li3—Li5	102.23 (3)	Si2^xiii^—Li7—Si2^xvi^	107.79 (3)
Li6A^xii^—Li3—Li5	164.79 (5)	Li5^xiii^—Li7—Li2^v^	72.37 (2)
Li6A^xiii^—Li3—Li5	76.55 (4)	Li6A^xiii^—Li7—Li2^v^	109.87 (3)
Li1—Li3—Li5^viii^	54.48 (2)	Si1—Li7—Li2^v^	97.64 (2)
Si2—Li3—Li5^viii^	57.97 (2)	Li4^v^—Li7—Li2^v^	68.809 (17)
Si2^xiii^—Li3—Li5^viii^	131.88 (2)	Li4—Li7—Li2^v^	156.71 (4)
Li4^xiii^—Li3—Li5^viii^	107.48 (3)	Si2^xiii^—Li7—Li2^v^	131.80 (3)
Li4—Li3—Li5^viii^	102.23 (3)	Si2^xvi^—Li7—Li2^v^	53.233 (8)
Li6A^xii^—Li3—Li5^viii^	76.55 (4)	Li5^xiii^—Li7—Li2	72.37 (2)
Li6A^xiii^—Li3—Li5^viii^	164.79 (5)	Li6A^xiii^—Li7—Li2	109.87 (3)
Li5—Li3—Li5^viii^	95.40 (4)	Si1—Li7—Li2	97.64 (2)
Li1—Li3—Li6B	98.86 (11)	Li4^v^—Li7—Li2	156.71 (4)
Si2—Li3—Li6B	56.17 (14)	Li4—Li7—Li2	68.810 (17)
Si2^xiii^—Li3—Li6B	96.70 (11)	Si2^xiii^—Li7—Li2	53.233 (8)
Li4^xiii^—Li3—Li6B	132.00 (14)	Si2^xvi^—Li7—Li2	131.80 (3)
Li4—Li3—Li6B	56.51 (11)	Li2^v^—Li7—Li2	103.06 (3)
Li6A^xii^—Li3—Li6B	147.86 (12)	Li5^xiii^—Li7—Li6B	135.66 (19)
Li6A^xiii^—Li3—Li6B	69.93 (11)	Li6A^xiii^—Li7—Li6B	73.41 (15)
Li5—Li3—Li6B	47.28 (10)	Si1—Li7—Li6B	61.14 (18)
Li5^viii^—Li3—Li6B	114.13 (14)	Li4^v^—Li7—Li6B	57.84 (5)
Li1—Li3—Li6B^viii^	98.86 (11)	Li4—Li7—Li6B	57.84 (5)
Si2—Li3—Li6B^viii^	56.17 (14)	Si2^xiii^—Li7—Li6B	96.57 (11)
Si2^xiii^—Li3—Li6B^viii^	96.70 (11)	Si2^xvi^—Li7—Li6B	96.57 (11)
Li4^xiii^—Li3—Li6B^viii^	132.00 (14)	Li2^v^—Li7—Li6B	126.62 (4)
Li4—Li3—Li6B^viii^	56.51 (11)	Li2—Li7—Li6B	126.62 (4)
Li6A^xii^—Li3—Li6B^viii^	69.93 (11)	Li5^xiii^—Li7—Li6B^xiii^	50.66 (18)
Li6A^xiii^—Li3—Li6B^viii^	147.86 (12)	Li6A^xiii^—Li7—Li6B^xiii^	11.59 (14)
Li5—Li3—Li6B^viii^	114.13 (14)	Si1—Li7—Li6B^xiii^	146.14 (18)
Li5^viii^—Li3—Li6B^viii^	47.28 (10)	Li4^v^—Li7—Li6B^xiii^	99.96 (10)
Li6B—Li3—Li6B^viii^	94.6 (3)	Li4—Li7—Li6B^xiii^	99.96 (10)
Li1—Li3—Li6B^xii^	112.27 (10)	Si2^xiii^—Li7—Li6B^xiii^	55.54 (4)
Si2—Li3—Li6B^xii^	134.21 (13)	Si2^xvi^—Li7—Li6B^xiii^	55.54 (4)
Si2^xiii^—Li3—Li6B^xii^	54.31 (10)	Li2^v^—Li7—Li6B^xiii^	103.19 (11)
Li4^xiii^—Li3—Li6B^xii^	55.03 (13)	Li2—Li7—Li6B^xiii^	103.19 (11)
Li4—Li3—Li6B^xii^	96.40 (11)	Li6B—Li7—Li6B^xiii^	85.00 (3)
Li6A^xii^—Li3—Li6B^xii^	9.86 (10)	Li5^xiii^—Li7—Li5^iii^	106.63 (4)
Li6A^xiii^—Li3—Li6B^xii^	98.97 (16)	Li6A^xiii^—Li7—Li5^iii^	168.88 (6)
Li5—Li3—Li6B^xii^	160.95 (12)	Si1—Li7—Li5^iii^	56.57 (2)
Li5^viii^—Li3—Li6B^xii^	84.41 (12)	Li4^v^—Li7—Li5^iii^	93.01 (3)
Li6B—Li3—Li6B^xii^	148.83 (4)	Li4—Li7—Li5^iii^	93.02 (3)
Li6B^viii^—Li3—Li6B^xii^	79.609 (14)	Si2^xiii^—Li7—Li5^iii^	117.29 (2)
Li3^ii^—Li4—Li3	107.43 (3)	Si2^xvi^—Li7—Li5^iii^	117.29 (2)
Li3^ii^—Li4—Li6B	72.1 (2)	Li2^v^—Li7—Li5^iii^	64.282 (19)
Li3—Li4—Li6B	68.24 (17)	Li2—Li7—Li5^iii^	64.281 (19)
Li3^ii^—Li4—Li6B^viii^	72.1 (2)	Li6B—Li7—Li5^iii^	117.71 (18)
Li3—Li4—Li6B^viii^	68.24 (17)	Li6B^xiii^—Li7—Li5^iii^	157.29 (18)
Li6B—Li4—Li6B^viii^	109.86 (19)		

Symmetry codes: (i) −*x*+1, −*y*+1, −*z*+1; (ii) *x*+1/2, −*y*+1/2, *z*; (iii) −*x*+1/2, *y*+1/2, −*z*+1; (iv) −*x*+1/2, *y*+1/2, −*z*; (v) *x*, *y*, *z*+1; (vi) *x*+1/2, −*y*+1/2, *z*+1; (vii) −*x*+1/2, *y*−1/2, −*z*; (viii) *x*, *y*, *z*−1; (ix) *x*+1/2, −*y*+1/2, *z*−1; (x) −*x*, −*y*, −*z*; (xi) −*x*+1/2, *y*−1/2, −*z*+1; (xii) *x*−1/2, −*y*+1/2, *z*−1; (xiii) *x*−1/2, −*y*+1/2, *z*; (xiv) −*x*, −*y*+1, −*z*+1; (xv) −*x*, −*y*+1, −*z*; (xvi) *x*−1/2, −*y*+1/2, *z*+1.

## References

[bb1] Brandenburg, K. (2012). *DIAMOND* Crystal Impact GbR, Bonn, Germany.

[bb2] Bruker (2008). *APEX2*, *SAINT* and *SADABS* Bruker AXS Inc., Madison, Wisconsin, USA.

[bb3] Chevrier, V. L., Zwanziger, J. W. & Dahn, J. R. (2010). *J. Alloys Compd*, **496**, 25–36.

[bb4] Frank, U., Müller, W. & Schäfer, H. (1975). *Z. Naturforsch. Teil B*, **30**, 10–13.

[bb5] Key, B., Bhattacharyya, R., Morcrette, M., Seznec, V., Tarascon, J. M. & Grey, C. P. (2009). *J. Am. Chem. Soc.* **131**, 9239–9249. 10.1021/ja8086278 19298062

[bb6] Key, B., Morcrette, M., Tarascon, J. M. & Grey, C. P. (2011). *J. Am. Chem. Soc.* **133**, 503–512. 10.1021/ja108085d 21171582

[bb7] Limthongkul, P., Jang, Y. I., Dudney, N. J. & Chiang, Y. M. (2003). *Acta Mater.* **51**, 1103–1113.

[bb8] Obrovac, M. N. & Christensen, L. (2004). *Electrochem. Solid State Lett.* **7**, A93–A96.

[bb9] Sheldrick, G. M. (2008). *Acta Cryst.* A**64**, 112–122. 10.1107/S0108767307043930 18156677

[bb10] Thomas, D., Abdel-Hafiez, M., Gruber, T., Huttl, R., Seidel, J., Wolter, A. U. B., Buchner, B., Kortus, J. & Mertens, F. J. (2013). *J. Chem. Thermodyn.* **64**, 205–225.

[bb11] Wang, P., Kozlov, A., Thomas, D., Mertens, F. & Schmid-Fetzer, R. (2013). *Intermetallics*, **42**, 137–145.

[bb12] Westrip, S. P. (2010). *J. Appl. Cryst.* **43**, 920–925.

[bb13] Zeilinger, M., Baran, V., Häussermann, U. & Fässler, T. F. (2013). *Chem. Mater.* **25**, 4113–4121.

[bb14] Zeilinger, M., Benson, D., Häussermann, U. & Fässler, T. F. (2013). *Chem. Mater.* **25**, 1960–1967.

[bb15] Zeilinger, M., Kurylyshyn, I. M., Häussermann, U. & Fässler, T. F. (2013). *Chem. Mater.*10.1021/cm4029885.

